# Aspects of the Behavior and Activity Rhythms of *Rowlandius potiguar* (Schizomida: Hubbardiidae)

**DOI:** 10.1371/journal.pone.0091913

**Published:** 2014-03-18

**Authors:** Marcus Paulo Alves de Oliveira, Rodrigo Lopes Ferreira

**Affiliations:** Laboratório de Ecologia Subterrânea, Departamento de Biologia/Setor de Zoologia, Universidade Federal de Lavras, Lavras, Brasil; Pennsylvania State University, United States of America

## Abstract

Although organisms of the order Schizomida are not widely distributed in caves throughout the world, they can, eventually, be abundant in certain regions, becoming a major faunal element in some caves. The majority of works on this order includes species descriptions, with rare references to behavioral aspects. As such, the present study describes the behavioral repertoire, and the activity and feeding periods of *Rowlandius potiguar* (Schizomida: Hubbardiidae) in the laboratory. The specimens were maintained in a terrarium, in an aphotic room, with temperature and humidity levels similar to the cave of origin. We used the focal-animal and *ad libitum* methods to describe behavior with qualitative and quantitative evaluations of behavioral acts. We witnessed nineteen behavioral acts, which is considered representative for observations in captivity. Two activity periods were observed: between 10 a.m. and 4 p.m. and between 10 p.m. and 4 a.m., characterizing an ultradian rhythm. In adaptive terms, this condition may be important for population maintenance in oligotrophic environments such as caverns. Necrophagy and cannibalism were also registered and could have been selected in the subterranean environment due to oligotrophy. The observation of rare and unprecedented behavior in this group, as well as the presence of rhythmicity in activity patterns, contribute to a better understanding of the ecological aspects of the species of this still little known Order.

## Introduction

The cave environment presents peculiar characteristics when compared to the epigean environment. Among them stand out the permanent absence of light and the tendency towards the stability of environmental conditions, such as temperature and humidity [1]. These conditions promote the establishment of a characteristic fauna, with species often specialized in subterranean life [1], [2].

Studies show that many species that inhabit the subterranean environment exhibit behavior different from those who live in the epigean. Hoenen and Gnaspini [2] found periods of circadian activity in species of epigean opiliones and alterations in the rhythms in a cave species. A similar result was found in the cricket species *Strinatia brevipennis,* from a cave in Brazil, in which alterations in the expression pattern of the circadian rhythm were detected [3].

Organisms of the order Schizomida are relatively frequent in the edaphic fauna of tropical and subtropical forests, generally being located in the upper soil layer (0–7 cm), among decaying leaves and twigs [4], [5]. However, it can also be found under stones, in termite mounds, rotten logs and caves [5], [6], [7].

The order Schizomida is currently represented by 258 species in 46 genera [8]. The fauna of Schizomida, in Brazil, is relatively little known [9]. Only eleven species have been registered for the country [7], [8], [10], one being introduced to the coast of Rio de Janeiro [11] and the others restricted to locals of the Amazon forest and Northeast Brazil [7], [12], [13]. However, numerous new species have been discovered in Brazil, suggesting that the number of described species results from the low sampling that historically has occurred in the country. As Schizomida species often have restricted distribution, it is expected that various new species are discovered and described with the increase of sampling sites [9].

Most articles on the order Schizomida are made up of species descriptions. There are some references to behavioral aspects, mainly feeding and reproduction, but there still are few references about behaviors exhibited by free ranging specimens (hereafter called the spontaneous behaviors) of this order [14], [15], [16].

Since the knowledge of the spontaneous behavior of individuals in order Schizomida is still incipient, especially when considering cave populations, the present study aimed to describe the behavioral repertoire of *Rowlandius potiguar* (Schizomida: Hubbardiidae) in the laboratory. The period of activity and feeding behavior of this species were also described.

## Materials and Methods

**Figure 1:**
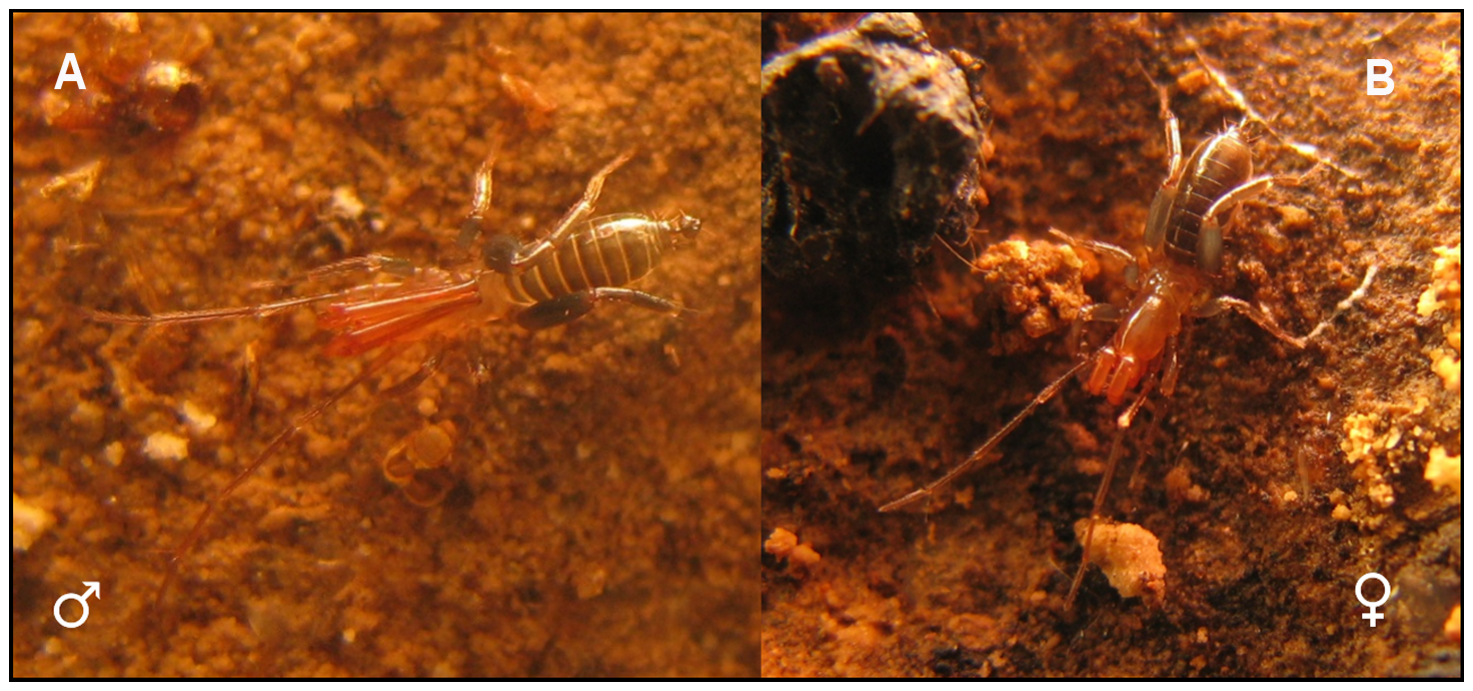
Specimens of *Rowlandius potiguar* in caves of Rio Grande do Norte, Brasil. A) Adult male heteromorph with long pedipalps and bulbous flagellum. B) Adult female with filiform flagellum.

The species *Rowlandius potiguar,* is distributed in 20 carbonate caves in the western area of the state of Rio Grande do Norte, Brazil [6], [7]. In some of these caves, especially those with high humidity, large populations are established. The most evident dimorphism is the flagellum, that is bulbous in males and filiform in females (Figure 1). This species also presents a notable dimorphism in pedipalp length in males, which are differentiated in heteromorphs and homeomorphs [7].

Specimens of the study were collected in the “Gruta do Geílson” limestone cave, located in the municipality of Felipe Guerra, Rio Grande do Norte, northeastern Brazil (5°35′53.23″ S, 37°41′17.52″ W).This cave is located in the karst area of the Apodi Group [17], inserted in the Caatinga [6]. It is characterized by the presence of two overlapping halls connected by a large fissure, apparently conditioned by a diaclasis. The only entrance to the cave is a small opening which allows the entry of organic material of plant origin, which accumulates near the entrance [6].The city has a semiarid climate, with rainy season from February to May, average annual temperature of around 27.8°C and annual relative humidity of 68% [18]. In this cave, the temperature remains similar to the annual average for the county throughout the year, though the humidity is higher, close to 90%. In a biospeleological survey conducted by Ferreira and colleagues [6], the presence of a large population of *Rowlandius potiguar* (over 200 individuals) was verified in this cave. Field collecting permits were issued to R.L. Ferreira by SISBIO/CECAV (license n. 14783-1). Cave geographic coordinates were obtained through CECAV's database (available at http://www.icmbio.gov.br/cecav/downloads/mapas.html).

For this study, 54 individuals were individually captured in three collection events: July 2008 (12 ind.), January 2009 (8 ind.) and July 2009 (34 ind.). All specimens were collected in the second hall of the cavity in an area with permanent absence of light and with high humidity. In 3 days, the specimens were transported to the Cave Ecology laboratory at the Federal University of Lavras, Lavras, Minas Gerais, Brazil. During transportation the organisms were individually maintained in plastic containers with moist paper towels, placed into Styrofoam boxes to avoid desiccation and sudden temperature changes.

**Figure 2:**
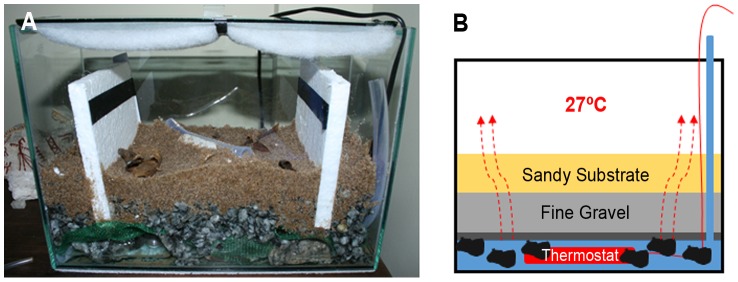
General aspect of the terrarium. A) 50 cm long, 15 cm wide and 20 cm in height, consisting of a layer of sandy substrate 4 cm thick and 4 cm of fine gravel. B) Schematic illustration of the temperature and humidity maintenance of the terrarium.

In the laboratory, the specimens were housed in a terrarium in a room with constant darkness (Figure 2A). The terrarium consisted of a 4 cm thick layer of sandy substrate (brought from the “Gruta do Geilson” cave) and 4 cm of fine gravel. At the base, a layer of rock supported the entire structure. Wood and leaf fragments, that served as shelter for the organisms, were placed on the substrate.

The terrarium was modified so as to maintain the temperature at about 27°C and humidity at around 90% (conditions similar to those observed in the cave) (Figure 2B). On the base of the terrarium, together with the supporting rocks, a two inch deep layer of water was maintained, which was heated by a thermostat and the temperature in the upper levels remained high, by convection (27±0.5°C). Heating the water also promoted the formation of vapor that condensed on the terrarium walls due to the lower external temperature of the environment, always maintaining the terrarium with high humidity (about 90%). The terrarium was monitored daily to evaluate these indices.

For individual or quantitative evaluations the specimens were held in individual transparent containers (11cm long, 4cm wide and 5 cm high). The base of the inner portion of these containers was coated by sandy substrate and the opening was closed with moist cotton. Humidity and temperature were maintained simulating the conditions prevalent in the original habitat of the species (90% and 27°C respectively). Specimens were kept in a room with permanent absence of light.

The feeding of the specimens was performed every two weeks by offering prey such as springtails and mites, which were captured and placed still alive in the individual containers and in the terrarium. In addition, the specimens also fed on small dipterans, symphylans, aphids and small millipedes, which were offered on some occasions.

The measurements were expressed as the amount of time (seconds) that the analyzed organism remained in that behavior. As a complementary method for the observation of group behavior (for the specimens placed in the terrarium) the *ad libitum* observation method [19] was used, which is a method without restriction as to what is being recorded and the observation period, allowing opportunistic observations. This method is effective in recording rare events and therefore significant for the behavioral study [20]. These observations were conducted with the presence of the observer (always the same person), all behaviors being recorded.

During the observations a red light was utilized to minimize the effects of artificial illumination on the animals (Startec luminaire, 10 lux, 680 nm) [3]. It was positioned 20 cm above the individual containers. For Schizomida, under the red light the specimens continued their regular behavioral repertoire, unlike when exposed to white fluorescent light, under which the individuals quickly sought shelter. It is well accepted among arachnologists that red light does not affect arachnid behavior [2].

Observations were made daily for two hours during two weeks (August 2008), describing the behavioral repertoire of the individuals. The quantification considered all behavioral acts shown, even those seen only once in a single individual.

The quantitative observations were conducted between August 2008 and November 2009. These observations were individualized, each individual being observed for a period of two hours daily (named “Individual quantitative observations”), taking turns with the times of day at which the same individual was observed in order to total a 24 hour observation per individual. Thus, the different behavior could be quantified in each individual during the morning, afternoon and evening shifts. We measure the time spent in each behavior by means of a stopwatch.

During individual observations, in addition to quantifying the duration of each behavioral act, the description of the feeding process was also conducted. Eating behavior was assessed immediately after the release of the prey inside each individual containers. Every movement made by the individual after the placement of the prey into the container was described (but not quantified).

The activity pattern of the specimens was determined as the sum of the values of all behaviors observed and measured for all individuals, excluding only the periods of immobility, during individual quantitative observations. We thus defined the active behavioral pattern through the percentage value of the activities measured during the observation periods. Between groups, we compared the behavioral patterns for nine females and six juveniles. Males were not evaluated due to the low number sampled (two specimens). To statistically test the activity pattern found, the periods of greater and lesser activity were grouped. The data of all individuals for each observation period (in seconds) were transformed into log x+1 and compared by the t test. The t test was also used to assess differences in activity patterns between females and juveniles. These analysis were carried out using the BioEstat 5.0 statistical software [21], and the results were considered significant when p<0.05.

We used linear regression among the different behavioral acts performed by the specimens in order to demonstrate how much each behavioral act influenced the occurrence of another behavior. This statistical analysis was conducted using R software model 2.15.3 [22]. The results were considered significant when p<0.05.

## Results

19 behavioral acts were seen for *Rowlandius potiguar*, which were grouped into six categories (Table 1). These acts were not observed in all individuals, this number of behavioral acts referring to the sum of the 17 subjects studied.

**Table 1:** Description of behavioral acts (in italics) and frequency (in %) of time spent in each act by specimens of Rowlandius potiguar observed in the different behavioral categories (in bold).

BEHAVIORAL ACT		DESCRIPTION	TOTALS (%)	MALES (%)	FEMALES (%)	JUVENILES (%)
**Exploration of the environment**		-	2.75	1.95	3.37	2.17
	*Walking*	Walking slowly through the area using the anteniforme pair of legs to probe the substrate.	*1.90*	*1.55*	*2.14*	*1.68*
	*Moving Leg1 (Anteniforme leg)*	Raise Leg 1 and perform random movements.	*0.01*	*0.07*	*0.02*	*0.00*
	*Moving Leg 3*	Raise Leg 3 and perform random movements.	*0.00*	*-*	*0.00*	*-*
	*Moving Pedipalps*	Repeatedly moving pedipalps while remaining motionless.	*0.69*	*0.21*	*1.05*	*0.35*
	*Groping Substrate*	Using the pair of anteniformes legs to probe the environment while remaining motionless.	*0.12*	*0.12*	*0.16*	*0.08*
	*Moving Abdomen*	Raise and lower the abdomen repeatedly while remaining motionless.	*0.03*	*-*	*0.00*	*0.06*
**Cleaning**		-	0.75	0.57	1.06	0.41
	*Clean Leg 1 (Anteniforme leg)*	Take Leg 1 in the chelicerae, cleaning it.	*0.28*	*0.13*	*0.41*	*0.15*
	*Clean Leg 2*	Take Leg 2 in the chelicerae, cleaning it.	*0.20*	*0.11*	*0.31*	*0.10*
	*Clean Leg 3*	Take Leg 3 in the chelicerae, cleaning it.	*0.09*	*0.06*	*0.12*	*0.05*
	*Clean Leg 4*	Take Leg 4 in the chelicerae, cleaning it.	*0.15*	*0.27*	*0.20*	*0.08*
	*Clean Pedipalps*	Repeatedly move pedipalps after ingestion of prey.	*0.02*	*-*	*0.00*	*0.03*
	*Clean Abdomen*	Raise the abdomen and rub the pedipalps on the ventral part, cleaning it.	*0.01*	*-*	*0.02*	*0.00*
**Avoidance**		-	0.01	*-*	0.02	0.01
	*Move backwards*	Walking slowly backwards with the pedipalps and pair of anteniformes legs extended.	*0.01*	*-*	*0.01*	*0.01*
	*Rapid movements*	Move rapidly forward	*0.00*	*-*	*0.01*	*0.00*
**Immobility**		-	96.44	97.48	95.48	97.38
	*Immobile*	Staying motionless with pedipalp retracted.	*94.96*	*97.48*	*95.46*	*94.09*
	*Extended pedipalp*	Staying motionless with pedipalp extended.	*0.01*	*-*	*0.02*	*0.00*
	*Buried*	Staying motionless under the substrate in the shelter.	*1.47*	*-*	*-*	*3.29*
**Hydration**		-	0.03	*-*	0.05	0.01
	*Active hydration*	Collect water droplets on the wall of the container with the pair of anteniformes legs and bring to the chelicerae.	*0.03*	*-*	*0.05*	*0.01*
Unknown movement		-	0.02	*-*	0.03	0.02
	*Unknown movement*	Performing various random movements.	*0.02*	*-*	*0.03*	*0.02*

The hyphen (-) indicates the absence of a behavior. The 0.00 represents low values not visible in two decimal places.

The 24-hour cycle of observations was completed with only 12 specimens (six females and six juveniles), the others having died before the end of the cycle of observations. No male completed the 24h series, so that the data relating to this group is restricted. Females showed longer survival in captivity, living up to 378 days (mean - ± SD  =  242±105 days), followed by juveniles (207±111 days). The male with longest time under laboratory conditions survived only 28 days (14±8 days).

The most frequent behavior was to remain motionless (Table 1). The males showed a longer period of immobility, followed by females and juveniles. For all groups, in the periods of mobility, the main active behavior was walking, followed by pedipalp cleaning and movement behaviors.

The predatory and feeding behavior of this species is characterized by a set of behavioral acts. The potential prey is readily perceived, by mechanisms not yet elucidated, immediately after its introduction into the enclosure of the specimens, but apparently the anteniforme pair of legs (the first pair of legs) is of great importance in this process, since the movement of the pair intensifies in these situations. When getting closer to the prey, the specimen poses in an attack/defense position, remaining motionless for a few seconds with the anteniforme legs and pedipalps open. If the prey escapes, a new search is performed on the substrate, when prey remains motionless, the specimens conduct their assault. The attacks are performed successively until successful or until the prey is no longer perceived in the environment.

In the immobility period preceding the assault, the organisms apparently evaluate the viability of capturing prey. If it has a larger size than the individual, there is no attack. However, if its size is favorable to attack (generally of similar or smaller size), there is an abrupt and quick approach, using the pedipalps and the pair of anteniforme legs to immobilize the prey. After capture, the prey begins to be torn into small fragments via the chelicerae.

The prey offered were generally smaller than the schizomida, reaching a maximum of 75% of its body size. Only for juvenile individuals was this percentage close to 100% and, on these occasions, such specimens invested in trying to catch the smaller prey, when present.

During feeding, the specimens perform movements with the pedipalps to change the orientation of the prey in relation to the chelicerae. The pairs of Legs 3 and 4 remain motionless, serving to support and lift. The second pair, in particular, is most often used to manipulate the food, while anteniforme pair (Pair 1) remains on the substrate most of the time and is used for faster and more complex movements such as changing the orientation of the prey in relation to the body of the specimen. At the end of the feeding process, it is generally not possible to see the remains.

After feeding, the specimens clean their pedipalps, taking them to the chelicerae, rubbing them against each other or by using the second pair of legs. The anteniforme legs and the first locomotor pair (second pair of legs) are also taken up to the chelicerae to be cleaned. The feeding process is followed by long periods of immobility in which activity can not be observed for days.

At a certain opportunity, necrophagy was observed to occur when a specimen fed on a dead mosquito provided in the container. In the terrarium, we observed the occurrence of cannibalism of adult females on juvenile individuals on two occasions.

**Figure 3:**
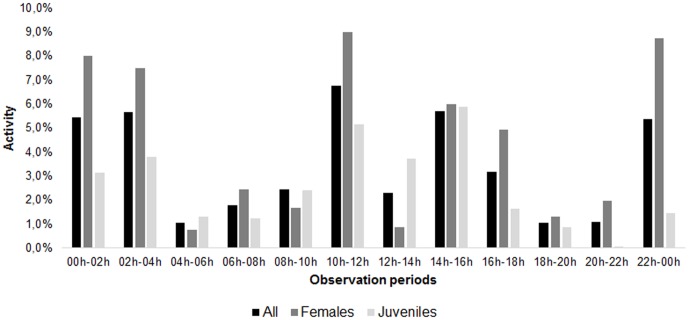
Percentages of activity presented by *Rowlandius potiguar* specimens in each observation period over 24 hour.

**Figure 4:**
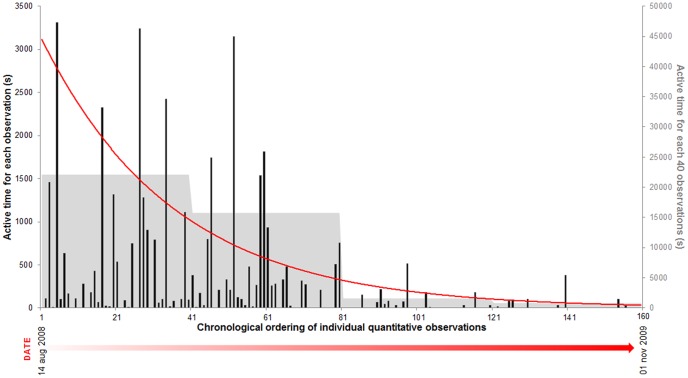
Total active time of *Rowlandius potiguar* specimens (17 individuals) in each individual observation during the study period. The X axis shows each individual observation in chronological order, from the beginning to the end of the study. The Y axis represents total active time during these observations. The dark bars represent the total active time during each individual observation. The gray bars in the background represent the total active time for every 40 observations. The red line represents the logarithmic correlation between total active time and individual observations (R^2^ = − 0.86).

The specimens had higher peak activity between 10 a.m. –12 p.m., followed by secondary peaks between 2 p.m. – 4 p.m., 10 p.m.- midnight, midnight - 2 a.m. and 2 a.m.- 4 a.m. (Figure3). The values found show two groups of high activity periods: the first, between 10 a.m and 4 p.m. and the other between 10 p.m and 4 a.m. The two groups of high activity were significantly different from periods of low activity (t = 2.099 and p = 0.04). Females and juveniles, when analyzed separately, showed a distinct pattern (Figure 3). Females activity periods followed the general pattern, with two groups of high activity (t = 1.998 and p = 0.02). However, such activity model is not applicable to juveniles (t = 1.398 and p = 0.08). During the period of the study, we observed an increasing reduction in the time for manifestation of active behavioral acts in individual containers (Figure 4).

**Figure 5:**
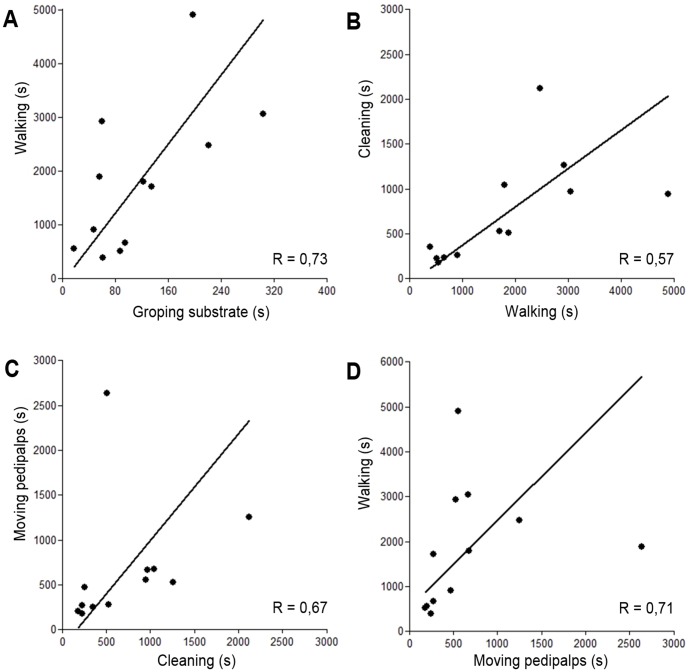
Positive correlations between main active behavioral acts. A) Positive correlation between “Groping the substrate” and “Walking” (p = 0.005). B) Positive correlation between “Walking” and “Cleaning Behaviors” (p = 0.001). C) Positive correlation between “Cleaning Behaviors” and “Moving pedipalps” (p = 0.001); D) Positive correlation between “Moving pedipalps” and “Walking” (p = 0.002).

We observed significant and positive relationships among the more frequent active behaviors (Figure 5), demonstrating how each behavioral act influences the occurrence of another behavior. After periods of immobility, the specimens usually initiated groping movement on the substrate. This behavior was followed by locomotion and later by cleaning activities. After cleaning, they usually conducted movement of the pedipalps, and soon after, again initiated movement, groping the substrate. This cyclical pattern of behavioral acts during periods of activity was frequently observed, interrupted only by periods of immobility and, more rarely, by other infrequent acts.

**Figure 6:**
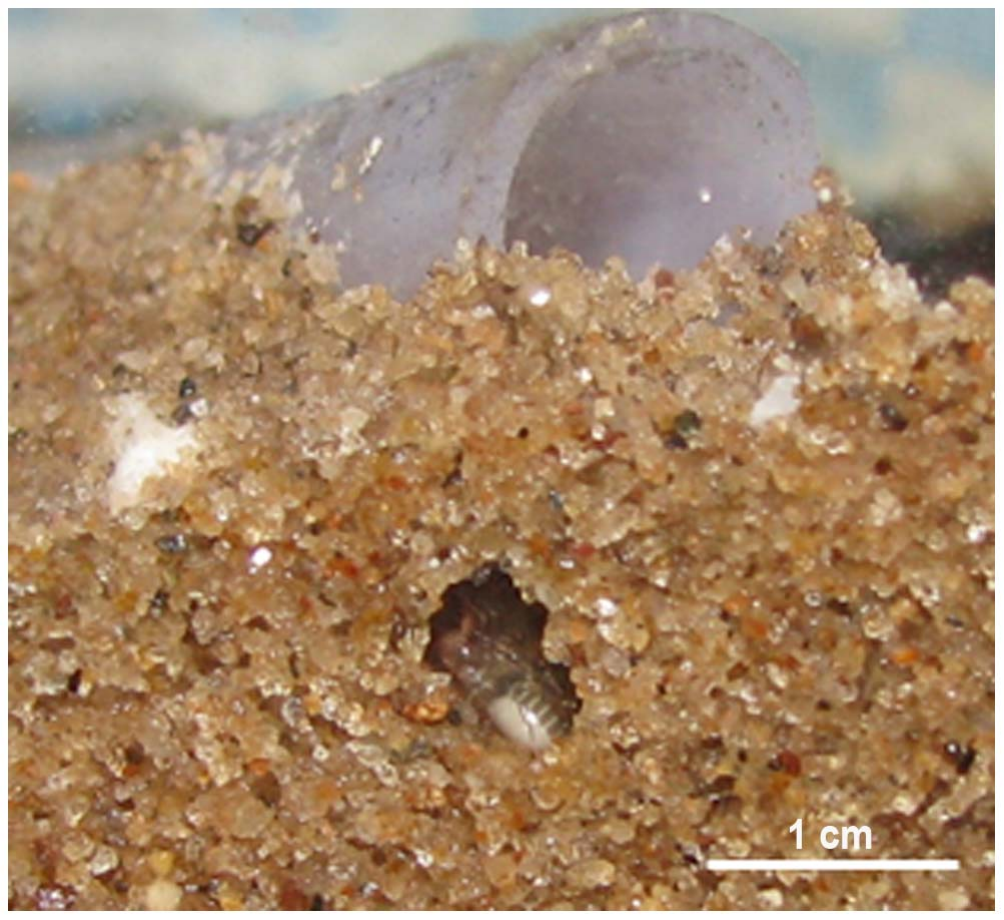
Example of nest built by a female specimen of *Rowlandius potiguar*.

Specimens often buried themselves under the terrarium substrate and in some individual subterranean shelters digged by themselves (Figure 6). These were preferentially located among the sand grains. The individuals generally used their shelters for long periods, staying buried for months. This behavior was recorded only for females and juveniles.

## Discussion

Works regarding the biology of the species of the order Schizomida are infrequent in the literature [16]. Humphreys and colleagues [15] addressed topics of nutrition, offspring and some habits of *Schizomus vinei*. Although Rowland [14] has studied the life history of a *Trithyreus pentapeltis* couple, addressing offspring behavior and juvenile development, spontaneous, dietary or those behaviors related to the pace of activities were not considered. Thus, it is impossible to perform the comparison of the behavioral repertoire exhibited by specimens of *Rowlandius potiguar* with other species of that order.

Other groups of arachnids exhibit wide variation in the number of behavioral acts. Ethograms made for species of Opiliones [23], scorpion [24] and pseudoscorpions [25] showed, respectively, 25, 17 and 95 behavioral acts. In the case of the species of scorpion (*Tityus serrulatus*), the low number of behavioral acts was related to the presence of parthenogenetic species, which were investigated solely from the female behavior point of view [24]. For the species of pseudoscorpion (*Paratemnoides nidificator*), the large behavioral repertoire is due to the social organization of the group, communication being the most representative behavior category, with 36 different behaviors based on the variations of the pedipalp vibrations [25]. Thus, as described by Lehner [26], the diversity of behavioral acts is related to social complexities of species [25]. As such, the 19 behavioral acts recorded in the present study are representative of observations in captivity, considering that this species has low social relationships and does not form aggregates of specimens.

The survival periods presented for specimens of this genus in the laboratory differ from those reported by Rowland [14] and Humphreys [15]. In the *Trithyreus pentapeltis* couple, Rowland noted that the female survived for 167 days, while the male exceeded that number (the exact period is not described). The life span of juvenile individuals was not measured accurately, however, according to Rowland, the sub-adult period seems to be between two and three years [14]. Humphreys and colleagues [15] collected and kept alive in the laboratory, only female *Schizomus vinei*, which survived up to 196 days. The low survival rate of male individuals in this study may be related to the reproductive period, as noted by Eickstedt [27] for spiders of the genus *Phoneutria*. Generally, as adults, the male specimens of this group die after the last copulation due to being quite debilitated [27].

The behavior of remaining motionless was frequent in individuals in captivity. However, there are differences in this behavior in relation to age and sex of the individuals observed. The long time spent motionless and low locomotion activity in adult males may be a result of the low number of individuals and times analyzed for this group. It can also be related to the direction of their energy towards reproduction, avoiding spending it on other activities, since, as observed in this study, the survival time for adult males was low. Furthermore, the specimens studied may, eventually, be territorialist, thus justifying the low mobility of adult males, because all individuals observed in each terrarium occupied each shelter alone. Perhaps repugnatory or anal glands, which are ventrally located, can be used to maintain territoriality.

Moreover, the higher movement activity in females may be related to the necessity to inspect large areas in search of prey or even seeking males for breeding, since they are rarer, andin many species, even unknown [5]. The females probably also can become more active during periods of oogenesis, when they requiring a greater amount of better nutritional quality food, which aids in the egg production. Thus, it is possible that females move more frequently due to reproductive periods.

*Rowlandius potiguar* apparently is an active predator, it will search for prey, as evidenced by observations in captivity. The act of catching prey proved complex in relation to the recognition of prey (especially with regard to its verification), and in this period there is a risk of the prey not being captured, or even being a potential predator. Thus, for females and juveniles, the locomotion activities for seeking prey may become more intense because they are less robust, which may hinder encountering more compatible prey.

The higher manifestation of substrate groping and cleaning and movement of pedipalp behaviors was expected, since the activity of the studied specimens show high correlation among the active behavioral acts. The correlation of these acts suggests that, during exploration of the environment, cleaning activities are necessary for a better perception of the environment. Small openings, called uropygid pores, of unknown function, have been described in *R. potiguar* [7]. These pores are present in the flagellum, tarsi of Leg 1 and pedipalps [7]. These last two locations are the focus of the major behavioral acts of the studied specimens when they are not walking. Thus, cleaning and constant movement of these areas, especially after walking, suggest that such pores have some function related to the perception of the environment.

The eating habits of schizomidas are little known [16]. However, the preference for Collembola, as cited by Beck [28], could be proven. However, schizomidas also fed on other groups such as mites, dipterans and aphids. Humphreys [15] reported that the size of prey captured by individuals of the species *Schizomus vinei* ranged from 10% to 100% of the total length of the Schizomida itself. In the present study, the attacks on smaller prey probably favored the predation process, particularly for juvenile specimens, since smaller prey exhibit lower defense and predators of larger instars have higher predation capacity [29].

The manner in which the specimens captured and manipulated the prey was similar to that described in other studies [4], [15], [30], [31]. However, Adis et al. [4] reported that the forelegs can be used to determine the size of the potential prey, which was not observed in our study. The absence of this behavior is probably due to high mobility and agility of the groups offered as prey. Thus, the use of the forelegs to grope the prey could frighten them, preventing capture.

The fast movements presented by the studied individuals have an important role in the predation process. It can be inferred that the successful capture of collembolas depended on the speed at which the specimen effected the attack and the distance between predator and prey.

Cannibalism in the order Schizomida has already been reported [14]. On the occasion, under laboratory conditions, adult males preyed on some juvenile individuals. Humphreys [15] reported that an adult female, also in captivity, fed on another, of smaller size.

The necrophagy observed in a specimen under study had not yet been reported for Schizomida. However, Sandidge [32] noted, for spiders of the genus *Loxosceles*, a feeding preference for dead prey. In the analysis, for the three varieties of live and dead prey offered, all the preference for the dead was more than 81%. For Schizomida, such a situation would be ecologically important, since opportunists and necrophages have an advantage over the most selective predators, because their feeding needs are more easily met [32]. For cave populations, as that studied, this premise would be even more significant because of the oligotrophic conditions prevalent in these environments. Thus, necrophagy could be an important habit, which would be selective in caves.

The activity pattern values found suggest the presence of an activity cycle every 12 hours, characterizing an ultradian rhythm, which denotes biological activities that occur in physiological cycles of 20 hours or less [33], [34]. In the model proposed by Aschoff and Gerkema [35], it was demonstrated that ultradian rhythmicity is responsible for coordinating metabolic and behavioral processes in a series of simultaneous events with maximum efficiency through better temporal and spatial distribution of available resources. Such a model is applicable for the specimens studied, mainly because their populations are in cave oligotrophic environments. Thus, the maximum utilization of the resource is quite advantageous for better success in the occupation of these environments.

The ultradian rhythmicity in activity patterns were also observed in spiders of the family Lycosidae (*Lycosa tarentula*) in cycles of continuous darkness [36]. The authors argued that the fact that this species naturally presents nocturnal activity which was responsible for the occurrence of shorter and more frequent cycles throughout the whole period in which it was submitted to the continuous aphotic condition. Thus, it is likely that the ultradian rhythmicity found for *R. potiguar* is an adaptive response to the permanent aphotic condition of the cave in which this population is established. In epigean populations, which are subject to variations in light levels and periods, different activity cycles may eventually occur.

The cave environment is little studied from the chronobiological viewpoint, especially when considering the artropods [37]. In these environments, the most common oscillators are absent or do not repeat surface patterns, such as the temperature and light cycle. It has been suggested that, adaptively, it makes no sense to have rhythm in a cycle-poor environment [38]. However, the limited data available for different species obtained under distinct experimental conditions demonstrate the presence of cycles [2], [3], [39], [40].

The difference between the activity periods found between females and juveniles could be, because the latter, despite the presence of rhythmic oscillations of the clock, do not observe the expression of biological rhythms due to the immaturity of the effector systems involved [33], [34]. In females, for being adults, the active behavioral pattern occurs more incisively with the proposed model.

During the experiment, a significant decrease in the time spent to perform the active behavioral acts throughout the observation time was perceived (Figure 4). The same pattern was found for the Scorpion *Tityus serrulatus* in captivity [24]. At the time, Mineo and colleagues [24] suggested the adaptation to the enclosure and knowledge of the limitations of displacement as the main factors that promoted the decrease in activity. In addition, the simplicity of the habitat and the absence of conspecifics in the enclosure may have contributed to the reduction of activity in *R. potiguar*.

It was observed that this species of *Rowlandius* has the ability to dig its own nest in captivity. Such behavior, however, is apparently rare in natural environments in which the organisms prefer to shelter themselves among the decaying leaves and branches, or humus, or in termite mounds and rotten logs [4], [5]. This may be due to the high availability of shelter of favorable micro-habitats in the forest soil, which does not impose the need to dig nests for their protection. Even under laboratory conditions, the Schizomida preferably search for spaces in the substrate rather than dig shelters, probably because it is a physically taxing and time-consuming activity, since the size of the sand grains are proportionally large compared to the body size of schizomids. As they are smaller, nymphs are more likely to find natural shelter (e.g. under rocks, logs, fissures) or even in the containers in which they were kept in the laboratory. On the other hand, adults are larger and require bulkier natural shelters. Thus, in the laboratory, since the substrate is compressed, this group had difficulty taking shelter in fissures, causing them to build their own nests. In the caves, these organisms do not necessarily remained sheltered, having been observed countless times walking on the floor substrate. The few individuals observed outside the caves were under rocks. Fallen logs in the litter are often used by small invertebrates as shelter, food and reproduction sites [41]. Thus, the availability of shelters can influence the sheltering behavior.

The behavioral study of cave species is important for a better understanding of the influence of the environmental stability and absence of light on these organisms. For Schizomida such research is even more important since few studies highlight the biological aspects of this order. The observation of rare and unprecedented behavior in this group, such as cannibalism and necrophagy, as well as the presence of ultradian rhythmicity in the activity patterns, contribute to a better understanding of the ecological aspects related to organisms of this Order.

## References

[1] Culver DC (1982) Cave Life: Evolution and Ecology. Cambridge, Massachussets and London, England: Harvard University Press.189 pp.

[2] Hoenen S, Gnaspini P (1999) Activity rhythms and behavioral characterization of two epigean and one cavernicolous harvestmen (Arachnida, Opiliones, Gonyleptidae). Journal of Arachnology 27: 159–164.

[3] Hoenen S (2005) Circadian patterns in the activity of the Brazilian cave cricket *Strinatia brevipennis* (Ensifera: Phalangopsidae). Eur J Entomol 102: 663–668.

[4] Reddell JR, Cokendolpher JC (1995) Catalogue, bibliography and generic revision of the order Schizomida (Arachnida). Texas Memorial Museum: Speleological Monographs 4: 1–170.

[5] Adis J, Reddell J, Cokendolpher J, Morais JW (1999) Abundance and phenology of Schizomida (Arachnida) from a primary upland forest in Central Amazonia. Journal of Arachnology 27: 205–210.

[6] Ferreira RL, Prous X, Bernardi LFO, Souza-Silva M (2010) Fauna subterrânea do Estado do Rio Grande do Norte: caracterização e impactos. Revista Brasileira de Espeleologia – RBE 1(1): 25–51.

[7] Santos AJ, Ferreira RL, Buzatto BA (2013) Two new cave-dwelling species of the short-tailed whipscorpion genus *Rowlandius* (Arachnida: Schizomida: Hubbardiidae) from northeastern Brazil, with comments on male dimorphism. Plos One 8(5): 1–12. 10.1371/journal.pone.0063616 PMC3661548 23723989

[8] Harvey MS (2007) The smaller arachnid orders: diversity, descriptions and distributions from Linnaeus to the present (1758 to 2007). Zootaxa 1668: 363–380.

[9] Santos AJ, Dias SC, Brescovit AD, Santos PP (2008) The arachnid order Schizomida in the Brazilian Atlantic Forest: a new species of *Rowlandius* and new records of *Stenochrus portoricensis* (Schizomida: Hubbardiidae). Zootaxa 1850: 53–60.

[10] Bonaldo AB, Pinto-da-Rocha R (2007) A new species of *Surazomus* (Arachnida, Schizomida) from Brazilian oriental Amazonia. Revista Brasileira de Zoologia 24: 323–326.

[11] Tourinho ALM, Kury AB (1999) The Southernmost record of Schizomida in South America, first record of Schizomida for Rio de Janeiro and of *Stenochrus* Chamberlin, 1922 for Brazil (Arachnida, Schizomida, Hubbardiidae). Boletim do Museu Nacional, Zoologia 405: 1–6.

[12] Cokendolpher JC, Reddell JR (2000) New and rare Schizomida (Arachnida: Hubbardiidae) from South America. Amazoniana 16: 187–212.

[13] Reddell JR, Cokendolpher J (2002) Schizomida. In: Adis J (Ed.). Amazonian Arachnida and Myriapoda – Keys for the identification to classes, orders, families, some genera, and lists of known species. Sofia: Pensoft Publishers. Pp. 387–398.

[14] Rowland JM (1972) Brooding habits and early development of *Trithyreus pentapeltis* (Arachnida, Schizomida). Pan-Pacific Entomol 83: 69–74.

[15] Humphreys WF, Adams M, Vine B (1989) The biology of *Schizomus vinei* (Chelicerata: Schizomida) in the caves of Cape Range, Western Australia. J. Zool. (Lond.) 217: 177–201.

[16] Armas LF (2004) Arácnidos de República Dominicana. Palpigradi, Schizomida, Solifugae y Thelyphonida (Chelicerata: Arachnida). Revista Ibérica de Aracnología, Volume especial Monográfico 2: 3–63.

[17] Medeiros WE, De Sá EFJ, Medeiros VC, Lucena LRF (2001) Estrutura geológica do aqüífero Açu na Borda Sul da Bacia Potiguar entre Apodi e Upanema, RN. Convênio CAERN/FUNPEC/UFRN. Relatório Técnico.

[18] Idema – Instituto de Desenvolvimento Sustentável e Meio Ambiente do Rio Grande do Norte (2008) Perfil do seu município: Felipe Guerra. Available: http://www.idema.rn.gov.br/contentproducao/aplicacao/idema/socio_economicos/arquivos/Perfil%202008/Felipe%20Guerra.pdf. Accessed 2013 February 19.

[19] Altmman J (1974) Observational study of behaviour: sampling methods. Behaviour 49: 227–265. 4597405 10.1163/156853974x00534

[20] Martin P, Bateson P (1986) Measuring Behaviour – an introductory guide. New York: Cambridge University Press. 200p.

[21] Ayres M, Ayres Jr M, Ayres DL, dos Santos AS (2007) BioEstat 5.0 - Aplicações estatísticas nas áreas das ciências biológicas e médicas. Belém, Sociedade Civil Mamirauá, 364p.

[22] R Development Core Team (2012) R: a language and environment for statistical computing. R Foundation for Statistical Computing, Vienna, Austria. ISBN 3–900051–07–0. Avalilable: http://www.R-project.org. Accessed 2013 July.

[23] Elpino-Campos A, Pereira W, Del-Claro K, Machado G (2001) Behavioral repertory and notes on natural history of the Neotropical harvestman *Discocyrtus oliverioi* (Opiliones: Gonyleptidae). Bull. British Arachnol. Soc. 12: 144–150.

[24] Mineo MF, Franco-Assis GA, Del-Claro K (2003) Repertório comportamental do escorpião amarelo *Tityus serrulatus* Lutz & Mello 1922 (Scorpiones, Buthidae) em cativeiro. Rev. Bras. Zoociências 5: 23–31.

[25] Tizo-Pedroso E, Del-Claro K (2011) Is There Division of Labor in Cooperative Pseudoscorpions? An Analysis of the Behavioral Repertoire of a Tropical Species. Ethology 117: 498–507.

[26] Lehner PN (1996) Handbook of Ethological Methods, 2nd edn. Cambridge: Cambridge University Press. 672p.

[27] Eickstedt VRD (1994) Aranhas de importância médica no Brasil. In: Barraviera B. Venenos Animais: uma visão integrada. Rio de Janeiro: EPUC. Pp. 151–172.

[28] Beck L (1968) Sobre a biologia de alguns aracnídeos na floresta tropical da Reserva Ducke (I.N.P.A., Manaus/Brasil). Amazoniana 1: 247–250.

[29] Polys GA, Myers CA, Holt RD (1989) The ecology and evolution of intraguild predation: potencial competitors that eat each other. Annu. Rev. Ecol. Syst 20: 291–330.

[30] Kraus O, Beck L (1967) Taxonomie und Biologie von *Trithyreus brasiliensis* n. sp. (Arach.:Pedipalpi: Schizopeltidia). Senckenbergiana Biol 48: 401–405.

[31] Sturm H (1973) Zurethologie von *Thrithyreus sturmi* Kraus (Arachnida, Pedipalpi, Schizopeltidia). Z. Tierpsychol 33: 113–140.

[32] Sandidge JS (2003) Scavenging by brown recluse spiders. Nature 426: 30. 10.1038/426030a 14603305

[33] Page TL, Block GD (1980) Circadian rhythmicity in cockroaches: effects os early post-embryonic development and ageing. Physio. Entomol 5: 272–281.

[34] Page TL (1985) Clocks and circadian rhythms. In: Kerkut GA, Gilbert LI.. Comprehensive Insect Physiology, Biochemestry and Pharmacology Vol. 6. Oxford: Pergamon Press. Pp. 577–652.

[35] Aschoff J, Gerkema M (1985) On diversity and uniformity of ultradian rhythms. In: SchulzHLavie P (Eds.). Ultradian Rhythms in Physiology and Behavior. Berlin: Springer-Verlag. Pp. 321–334.

[36] Ortega J, Ruiz M, Fernandez-Montraveta C (1992) Daily Patterns of Locomotor Activity in a Lycosid Spider. Journal of Interdisiplinary Cycle Research 23: : 4 295–301.

[37] Marques N, Menna-Barreto L (1997) Cronobiologia. 1^st^ Ed. São Paulo: Editora Fiocruz. 328p.

[38] Saunders DS (1982) Insect Clocks. 2^nd^ Ed. Oxford: Pergamon Press. 409p.

[39] Lamprecht G, Weber F (1986) Time-keeping mechanisms and their ecological significance in cavernicoluous animals. NSS Bull 47: 147–162.

[40] Trajano E, Menna-Barreto L (1995) Locomotor activity patterns of Brazilian cave catfishes under constant darkness (Siluriformes: Pimelodidae). Biol. Rhythm Res 26: 341–353.

[41] Hamilton WD (1978) Evolution under bark. In: Mound LA, Waloff, E (Eds.)Diversity of insects faunas. Oxford: Blackwell Scientific Publications. Pp. 154–175.

